# Metabolic and Immunological Effects of Intermittent Fasting on a Ketogenic Diet Containing Medium-Chain Triglycerides in Healthy Dogs

**DOI:** 10.3389/fvets.2019.00480

**Published:** 2020-01-08

**Authors:** Y. Becca Leung, Nick J. Cave, Axel Heiser, Patrick J. B. Edwards, A. Jonathan R. Godfrey, Tim Wester

**Affiliations:** ^1^School of Veterinary Science, Massey University, Palmerston North, New Zealand; ^2^AgResearch, Grasslands Research Centre, Hopkirk Research Institute, Palmerston North, New Zealand; ^3^School of Fundamental Sciences, Massey University, Palmerston North, New Zealand; ^4^School of Agriculture and Environment, Massey University, Palmerston North, New Zealand

**Keywords:** fasting, ketone, beta-hydroxybutyrate, ketogenic, diet, dog, medium-chain, immunity

## Abstract

In several species, intermittent fasting (IF) has been shown to have beneficial effects, including delayed aging, increased lifespan, increased insulin sensitivity, reduced ischemic tissue damage, delayed onset of neurodegenerative disease and improved neuronal repair following injury. However, the metabolic and immunological effects of IF have not been well-established in dogs. The aim of this study was to examine the effects of a 48 h IF regimen using a low fat and a high fat diet in healthy dogs by quantifying the metabolic, hormonal, and immunological changes. We hypothesized that IF dogs would have higher blood ketone and ghrelin concentrations, lower blood leptin, insulin and glucose concentrations, and signs of immunosuppression compared to dogs eating daily. Ten healthy adult dogs were randomized into three group and underwent three feeding regimes in a 3 × 3 Latin square design: twice a day feeding on a low fat (23% energy from fat; LF) diet, 48 h fasting on a low fat diet, and 48 h fasting on a high fat enriched with medium-chain triglycerides (68% energy from fat; HF) diet. Body weight, food intake, activity, blood glucose, β-hydroxybutyrate, leptin, ghrelin, and insulin were measured. Lymphocyte proliferation and neutrophil/macrophage phagocytosis and respiratory burst were measured as markers of immune function. Nuclear magnetic resonance spectroscopy was used to relatively quantify plasma metabolites. When the dogs were IF on a HF diet, they had the highest concentration of blood ketones (mean 0.061 mmol/L, SD 0.024), whereas they had the lowest concentration (mean 0.018 mmol/L, SD 0.004) when fed daily. Blood glucose and insulin concentrations were lower in IF dogs on a HF diet compared to daily feeding or IF on a LF diet. There was an increase in plasma β-hydroxybutyrate concentrations, and a reduction in glucose and insulin concentrations when dogs were IF on a HF diet. There was only a decline in the immune parameters studied when the dogs were IF on a LF diet, which was not seen when on the HF diet. The results of this study indicate the potential of IF to be further investigated as a potential beneficial feeding regime for dogs.

## Introduction

Optimal feeding regimens for both veterinary and human hospitalized patients have not yet been established. Underfeeding is common during hospitalization and is associated with depressed immunity, increased readmission rates, and increased mortality (1–4). However, overfeeding critically ill patients has also been shown to have deleterious effects (5–7). In particular, iatrogenic hyperglycemia can lead to impaired wound healing, neuronal dysfunction, increased production of the inflammatory cytokines interleukin-6 (IL-6) and tumor necrosis factor-α (TNF-α), inhibition of leukocyte function, vasculitis, and ultimately a poorer clinical outcome (8–13). Some of these effects can be abrogated when blood glucose is normalized (9, 14). So whereas clinicians want to provide appropriate nutrition for hospital patients, they need to establish the best means of doing so, while avoiding hyperglycemia.

A potentially effective feeding regime to achieve these agnostic goals is intermittent fasting. Intermittent fasting is the process of reducing meal frequency in order to prolong the period of fasting between meals, but without necessarily restricting total caloric intake when expressed over a longer period of time. Extending the period of fasting between meals has been found to increase insulin sensitivity, reduce serum fructosamine, reduce cancer cell proliferation, reduce concentrations of pro-inflammatory cytokines IL-6, IL-1β, and TNF-α in circulation, delay aging, and improve neuronal repair following injury when compared to continuous feeding (15–25). In healthy mice, blood glucose and insulin concentrations were reduced following a period of intermittent fasting (21). Several mechanisms have been proposed including the reduction of mitochondria-derived reactive oxygen species, activation of sirtuins and associated promotion of autophagy and cell cleansing, and decreased expression of p38 mitogen-activated protein kinase, an upstream mediator of apoptosis (18, 26–28). These potential mechanisms would allow a reduction in oxidative stress and a more tailored repair response following injury.

An intermittent fasting regime is of particular interest in patients with spinal disease, as it has been shown to reduce lesion size and improve recovery in rodent models of spinal injury compared with daily feeding (21–23). Ketones, which increase during the fasting period, upregulate nicotinamide adenine dinucleotide (NAD)^+^-dependent sirtuin 3 and superoxide dismutase, increases the expression of autophagy-promoting protein forkhead box O3a, and reduces neuronal injury in the cerebral cortex of rats following experimentally induced hypoglycemia (29, 30). In addition, ghrelin, an orexigenic peptide secreted by the stomach in a fasted state, protects neurons from ischemia and reperfusion injury *in vivo*, and decreases the expression of the TNF-α and IL-1β from microglial cells in Parkinson-modeled mesencephalic neuronal cell cultures (31, 32). Thus, intermittent fasting may be a feeding strategy that promotes neuronal recovery while also avoiding hyperglycemia.

Although there are many potential benefits to intermittent fasting, there are possible detrimental consequences as well. Humoral and cellular immune functions are known to decrease in a fasted state, which is in part the result of a drop in the plasma adipokine, leptin (33, 34). Leptin increases neutrophil chemotaxis and macrophage phagocytosis, and affects the maturation of T-cells (35). Short-term fasting in several species reduces T-cell mediated responses, and splenic and peripheral immune cell numbers (17, 36, 37). Injection of leptin into fasted or leptin-deficient ob/ob mice reverses the suppression of lymphocyte differentiation, macrophage phagocytosis, and delayed-type hypersensitivity responses caused by leptin deficiency (34, 38, 39). However, rats consuming a high fat diet attenuates the drop in leptin during fasting, and increases ketone production between meals (40, 41). In addition, feeding medium-chain triglycerides (MCTs) promotes the formation of ketones in the fed state (42, 43). After eating, the majority of medium-chain fatty acids are absorbed through the portal circulation and are metabolized by hepatocytes into ketones (44, 45). So feeding a high fat diet enriched with MCTs may have the dual benefit of maintaining leptin serum concentration while also promoting ketogenesis during the short periods of fasting. Therefore, intermittent fasting on a high fat diet enriched with MCTs may be preferential.

The principal aim of this study was to determine the metabolic and immunological effects of a 48 h intermittent fasting regime in healthy dogs. Our primary hypothesis was that dogs undergoing intermittent fasting would have higher plasma concentrations of β-hydroxybutyrate and ghrelin, and lower concentrations of glucose, insulin, and leptin compared with dogs fed daily. Our secondary hypothesis was that dogs undergoing an intermittent fasting regime on a high fat diet enriched with medium-chain triglycerides would have a greater blood β-hydroxybutyrate and leptin concentrations compared with dogs intermittently fasted on a low fat diet.

## Materials and Methods

### Animals

Following a complete physical examination, 10 healthy, adult dogs from Massey University's Canine Nutrition Unit were used in this study. The dogs were of two breeds: Harrier Hounds (*n* = 7) and New Zealand Huntaways (*n* = 3), and were composed of four neutered males and six speyed females. The dogs had a mean age of 7.1 (SD 2.1) years, mean body weight of 27.8 (SD 3.1) kilograms, and a mean body condition score (BCS) of 4.2 (SD 0.4). The study protocol was approved by the Massey University Animal Ethics Committee (MUAEC #16/130).

### Study Design

A week before the commencement of the study, all dogs were transitioned onto a high carbohydrate, low fat commercial dry diet to allow for acclimation. The dogs were fed to meet their maintenance energy requirement based on historical colony data. After this acclimation period, the dogs were randomized into one of three groups which underwent each feeding trial regime in a 3 × 3 Latin-square design with a weeklong “wash out” duration in-between. The three feeding regimes were as follows: (1) daily fed feeding on a low fat (LF), high carbohydrate diet (BID), (2) intermittent fasting (feeding once every 48 h) on the same LF diet (IF LF), and (3) intermittent fasting (feeding once every 48 h) on a high fat (HF) diet (IF HF). Both diets used in this study were formulated to meet the nutrient requirements for adult dogs defined by the Association of American Feed Control Officials (AAFCO). A commercial dry food^1^ was chosen as the low-fat, high carbohydrate diet. The high fat diet was created using the same dry commercial diet with the addition of powdered whey protein, beef tallow, sunflower oil, coconut oil and a multivitamin/mineral mix^2^ to ensure adequacy of the total diet. The total amount of medium-chain triglycerides (C8, C10, C12) from the coconut oil and beef tallow amounted to 14.7% of the total calories in the diet when using an energy of 6.8 kcals/gram for the MCTs (46). The nutrient profiles of both diets are presented in Table 1.

**Table 1:** The nutrient profile of the low fat commercial diet and the modified high fat diet.

	**Commercial low fat diet**	**Modified high fat diet**	**Percentage difference (%)**
Protein energy (%ME)	22	22	100.00
Fat energy (%ME)	23	68	295.57
Carbohydrate energy (% ME)	55	10	18.19
Protein (g/Mcal)	62.79	62.95	100.26
Total lipid (g/Mcal)	26.91	79.41	295.08
Linoleic acid (18:2 n-6) (g/Mcal)	8.16	9.37	114.75
Carbohydrate (g/Mcal)	157.57	29.92	18.99
Choline (mg/Mcal)	717.60	670.54	93.44
Folate (mcg DFE/Mcal)	209.30	388.12	185.44
Niacin (mg/Mcal)	14.35	21.99	153.23
Pantothenic acid (mg/Mcal)	7.41	7.30	98.42
Riboflavin (mg/Mcal)	2.03	2.34	115.30
Thiamin (mg/Mcal)	1.11	0.70	63.56
Vitamin A (mcg RAE/Mcal)	4041.86	1301.85	32.21
Vitamin B-12 (mg/Mcal)	0.018	0.017	94.44
Vitamin B-6 (mg/Mcal)	2.09	1.17	56.09
Vitamin E (α-tocopherol) (IU/Mcal)	74.75	88.18	117.97
Calcium (g/Mcal)	2.84	3.13	110.39
Copper (mg/Mcal)	4.34	3.15	72.64
Iodine (mg/Mcal)	0.94	0.71	75.77
Iron (mg/Mcal)	45.71	34.39	75.24
Magnesium (g/Mcal)	0.32	0.27	85.31
Manganese (mg/Mcal)	15.03	4.55	30.29
Phosphorus (g/Mcal)	2.39	1.79	74.92
Potassium (g/Mcal)	2.21	2.59	116.90
Selenium (mg/Mcal)	0.14	0.10	69.23
Sodium (g/Mcal)	1.20	0.50	41.39
Zinc (mg/Mcal)	63.39	52.99	83.59
Vitamin D (IU/Mcal)	447.00	275.92	61.73

*The high fat diet was created using the commercial low fat diet with the addition of whey protein isolate, beef tallow, sunflower oil, coconut oil, and a multivitamin/mineral mix. Both diets were formulated to meet the nutrient requirements for adult dogs as defined by the Association of American Feed Control Officials (AAFCO)*.

When dogs were in the daily feeding regime, they were offered their maintenance energy requirement divided equally into two meals that were provided in the morning and the afternoon. When the dogs were in the intermittent fasting regime, they were offered twice their maintenance energy requirement in the morning every other day. The dogs were allowed up to 3 h to consume their meal, after which the food was removed and weighed. During the wash out period between feeding regimes, all dogs were placed on the commercial high carbohydrate, low fat dry diet and fed twice a day for 1 week.

On days 1, 3, 5, and 7 of a trial period, a fasted blood sample (12 mL in total) was collected into lithium heparin and plain red-top vacutainers^3^ from all dogs by jugular venipuncture before food was offered. Day 1 represented an overnight-fasted, baseline sample, while the samples collected on days 3, 5, and 7 represented either a 12 h fast when the dogs were eating daily, or a 48 h fast when the dogs were fasted intermittently. Immediately following blood collection, a protease inhibitor^4^ was added to the sample in the plain red-top vacutainer to prevent ghrelin degradation. All samples were placed on ice until they were centrifuged, and serum and plasma removed. Daily food intake, weekly body weight and body condition score were recorded for all dogs. To compare the caloric intake of the dogs on the different feeding regimes, the total calories eaten every 2 days (i.e., days 1 and 2, days 3 and 4, days 5 and 6) was divided by the weekly starting weight of each dog to the power of 0.75 in order to express intake as kcal per 48 h/kgBWT^0.75^. In addition, day (5 am to 8 pm) and night (8 pm to 5 am) activity of the dogs was measured using a tri-axial accelerometer^5^ fitted to their collar.

After blood sampling on day 7, all dogs were placed onto the “wash out” feeding regime. Following a wash out, each group was fed according to their next assigned feeding regime, and blood samples taken as described above. This was repeated again once more so that all groups underwent each of the three different feeding regimes with a washout period in between.

### β-Hydroxybutyrate, Glucose, and Metabolomics

Within 1 h of collection, plasma was harvested from the heparin vacutainers following centrifugation for 10 min at 3,000 rcf at 4°C. Plasma glucose was analyzed using a handheld glucometer^6^ which has been previously validated for use in dogs (47). The remaining plasma was stored at −80°C until further analysis.

β-hydroxybutyrate was assayed in plasma samples from days 3, 5, and 7 of each regime using a colorimetric assay^7^ according to the manufacturer's instructions. The thawed plasma samples were initially de-proteinated using 10 kD spin columns^8^ and centrifuged at 10,000 rcf for 10 min. The samples were then prepared and absorbance at 450 nm was measured using a microplate reader^9^.

Nuclear magnetic resonance (NMR) was used for plasma metabolomics. Samples were prepared based on the protocol described in a previous study (48). Briefly, 300 μL of plasma was deproteinated using 600 μL of methanol and incubated at −20°C for 30 min. The samples were then centrifuged at 13,400 rcf for 30 min and the supernatant was removed and placed in a rotary evaporator for 3.5 h at 20°C. Any remaining supernatant in the samples was then dried completely by freeze drying. The dried samples were then stored in screw top vials^10^ at −80°C until analysis. On the day of analysis, a phosphate buffer solution was prepared by dissolving 928.6 mg of anhydrous NaH_2_P0_4_ and 320.9 mg of anhydrous Na_2_HPO_4_ in 100 g of D_2_0, and used with further pH modification. The dried samples were reconstituted using 600 μL of phosphate buffered D_2_O, along with two standards [0.5 mM of 2,2-dimethyl-2-silapentane-5-sulfonate sodium salt (DSS)^11^ and 5 mM imidazole^12^] to allow for adjustments in chemical shifts and pH. The samples were then transferred to 5 mm NMR tubes^13^ and analyzed using a cyroprobe-equipped Bruker Avance 700 MHz spectrometer (Bruker Biospin, Rheinstetten, Germany) operating at 700.13 MHz. The samples were measured at 25°C using a standard 1D NOESY pulse sequence with presaturation of the residual water signal. Spectra were recorded using 58k points and an acquisition time of 3.5 s followed by a relaxation delay of 1.5 s. TopSpin v3.0 (Bruker Biospin) was used to process the ^1^H NMR spectra. Phasing and baseline correction of all NMR spectra were checked manually.

### Endocrinology

The plain red-top vacutainers were centrifuged for 10 min at 3,000 rcf at 4°C, and serum removed within 1 h of collection. The serum was stored at −80° C until analysis. Leptin, ghrelin, and insulin were assayed using a commercial multiplex immunoassay^14^. Samples were prepared following the manufacturer's instruction. Briefly, 50 μL of serum was added to a 96-well plate to which a buffer and antibody-conjugated beads were added. The plates were gently agitated overnight at 4°C. Following this, the plates were washed, detection antibodies added and finally analyzed using a multiplex reader^15^.

### Immunological Assays

**Figure 1:**
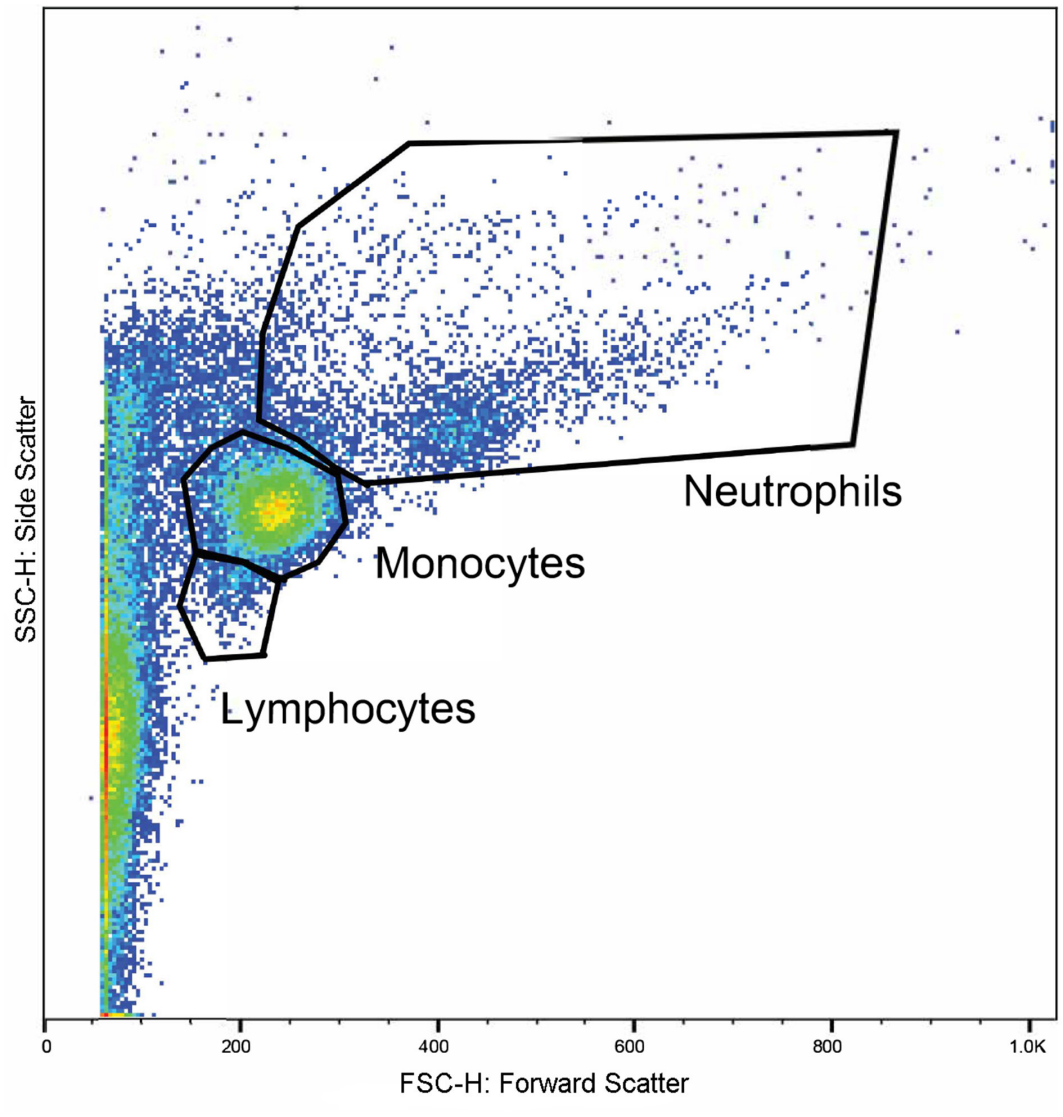
A representative plot displaying the flow cytometry scatter properties of leukocytes in one sample. The forward scatter (x-axis) corresponds to size of the cells and the side scatter (y-axis) corresponds to the cells' granularity. Regions were gated around clusters of cells corresponding to the expected locations for lymphocytes, monocytes, and neutrophils.

**Figure 2:**
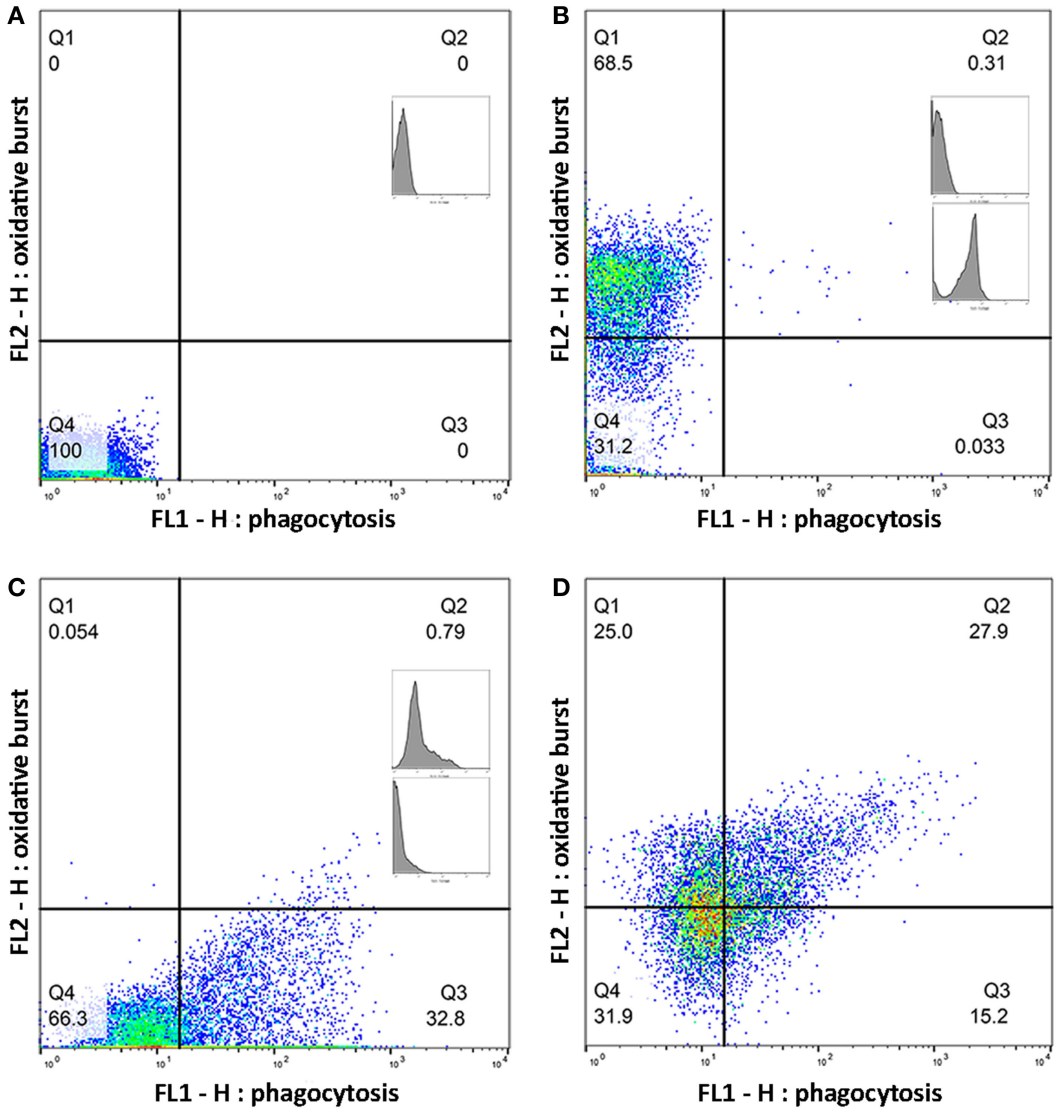
Quadrants for single and double positive cells were established using a set of control samples. Plots **(A–D)** show a representative sample of gated monocytes from one sample. The number in each quadrant is the percentage of cells. **(A)** Monocytes without a flurochrome added. **(B)** Monocytes with only dihydrorhodamine (DHR) 123 added. **(C)** Monocytes with only pHrodo™ Red *S*. *aureus* added. **(D)** Monocytes with both DHR and pHrodo™ Red *S. aureus* added.

Whole blood from the lithium vacutainers was utilized within an hour of collection. Leucocyte phagocytosis and respiratory burst were assayed by flow cytometry using a cell analyzer^16^ and fluorescent markers for phagocytosis^17^ and oxidative burst^18^. A modified protocol was used which was based on previously published works (49, 50). Four flow cytometry tubes were prepared for each sample. In each tube, 100 uL of whole blood was incubated at 37°C for 30 min with either 10 μL of 143 μM DHR solution, 50 μL of pHrodo™, both compounds, or nothing added. After incubation, 2.7 mL of deionized water was added to lyse erythrocytes. Within 2 min of adding the deionized water, 300 μL of 10X PBS was added and samples centrifuged at 600 rcf for 7 min. The supernatant was decanted, and the process repeated with 3 mL of PBS. Then, 3% formaldehyde in PBS was added and samples incubated at room temperature for 5 min for fixation. Finally, 2 mL of PBS was added, and samples were centrifuged at 350 rcf for 7 min. The supernatant was removed, and the pellet suspended in 200 μL PBS with 2% fetal calf serum (FCS) in 5 mL polystyrene round bottomed tubes^19^. Samples were acquired with a flow cytometer^20^ until at least 10,000 events were collected. Lymphocyte, monocyte, and neutrophil populations were identified based on size and granularity in a forward and side scatter plot (Figure 1). Quadrants for single and double positive cells were demarcated using the set of control samples (Figure 2). The results of the gated populations were expressed as the percentage positive for cells undergoing phagocytosis and/or respiratory burst, and their mean fluorescence intensity.

Lymphocyte proliferation was performed on heparinized whole blood. For each sample, 25 μL of blood was transferred into eight wells on a 96 U-well plate. Then, 200 ng/mL of *Staphylococcus* enterotoxin B (SEB)/lipopolysaccharides (LPS) solution was added to four of the wells. The plates were then incubated at 37°C in 5% CO_2_ humidified atmosphere for 3 days. Following this, 50 μL of ^3^H-thymidine of a 10 μCi/mL stock solution was added to each well. The plate was incubated for 4 h at 37°C in 5% CO_2_ humidified atmosphere for 4 h and then stored at −80°C until analysis. The cells were then harvested and counted using liquid scintillation.

### Sample Size

An a priori power analysis was performed using a desired mean difference and previously published standard deviations for key metabolites and hormones. The mean difference and standard deviation (SD) used in the power analysis were: β-hydroxybutyrate 0.05 (SD 0.01 mmol/L), ghrelin 75 (SD 53 pg/mL), leptin 3,000 (SD 3,000 pg/mL), and insulin 220 (SD 150 pg/mL). This indicated that a sample size of 10 dogs would be necessary for significance level (α) of 0.5 and a power of 80% to detect a difference in β-hydroxybutyrate, ghrelin, leptin, and insulin.

### Statistical Analysis

#### Metabolomics

For analysis, the NMR spectra were divided into 0.04 ppm spectral buckets, where the regions corresponding to water and DSS (4.68 to 4.88, −0.1 to 0.1 ppm, respectively) were excluded, along with the following additional regions 5.51 to 5.84, 5.92 to 6.07, and 6.11 to 6.31. All spectra were normalized by total intensity.

The relationship between the diet groups and the metabolome was explored using principal component analysis (PCA), partial least squares-discriminant analysis (PLS-DA) and orthogonal partial least squares discriminant (OPLS-DA) analysis were performed using SIMCA v13.0 (Umetrics, Sweden). These statistical methods can reveal clustering of samples into different groupings based on differences of metabolite concentrations across the sample population. PCA is an unsupervised method and is perhaps the most robust. PLS-DA and OPLS-DA are so-called supervised methods and use a priori knowledge of the group membership to fit the data and maximize separation of data from these groups. Pareto scaling was used for the supervised models and the PLS models were validated by permutation testing to rule out overfitting. The spectral buckets that contributed to the greatest variance in the samples were identified from loading plots and subsequently assigned to their associated metabolites using the Chenomx metabolite library v8.3 (Chenomx Inc., Alberta, Canada). The metabolite concentrations were then quantified using manual fitting of the spectral peaks in the Chenomx NMR Suite. Concentrations of plasma β-hydroxybutyrate measured by the colorimetric assay were compared to the concentrations obtained from the fitted spectra using “BlandAltmanLeh” v0.3.1 package (51) in the R Studio v1.1.456 statistical software (52).

#### Modeling

The “lme4” package (53) in the R Studio v1.1.456 statistical software (54) was used to perform a linear mixed effects analysis of the relationship between the outcome variables [change in weight, intake, activity, glucose, β-hydroxybutyrate, leptin, ghrelin, insulin, homeostatic model assessment (HOMA), NMR metabolites, lymphocyte proliferation and flow cytometry results], and the fixed variables (time, diet, age, sex, and BCS). Separate models were fitted for each outcome variable. Dog was included as a random effect to account for repeated measures. Interactions between the fixed variables, and between diet and diet sequence, were not significant, and so were not included in the final models.

If the visual inspection of the residual quantile-quantile plots and the Shapiro-Wilk test of the residuals indicated a deviation from normality or homoscedasticity, then transformations of the dependent variable were performed in attempts to improve consistency on the assumptions of the model. However, transformation did not lead to a change in the interpretation of the models or our conclusions. Therefore, for simplicity, the graphs, and final model are reported with the untransformed data.

A *post-hoc* pairwise comparison of the estimated marginal means with Tukey's correction was performed when diet was significant in the final model. The results of the mixed effects models are presented as the means and standard error of means. *P* ≤ 0.001 were considered indicative of very strong evidence, *p* ≤ 0.01 of strong evidence, *p* ≤ 0.05 of moderate evidence, *p* ≤ 0.1 of weak evidence, and *p* ≤ 0.1 of insufficient evidence (55).

## Results

### Intake, Body Weight, Body Condition Score, and Activity

There were no differences in any of the baseline parameters before the groups began their assigned feeding regime in any of the treatment weeks (*P* > 0.5). All dogs remained at a BCS of 4 or 5 out of 9 throughout the study. When fed daily, the dogs had a higher food intake compared to when they were fed intermittently (*P* < 0.001), however, there was no difference in intake between dogs when intermittently fed on the LF or the HF diets (*P* = 0.395, Table 2). Male dogs consumed more food per kgBWT^0.75^ than female dogs (mean 277 ± 67 vs. 226 ± 50 kcals/kgBWT^0.75^). The dogs lost more weight when intermittently fasted on a low fat diet, but there was no difference in the percentage of body weight change when the dogs were daily fed compared to when they were intermittently fasted on a high fat diet regime (Table 2). In addition, when the dogs were fed daily, they were more active at night compared to when they were intermittently fed the LF diet (*P* = 0.028) and HF diet (*P* = 0.012).

**Table 2:** The means and standard deviations of food intake, change in bodyweight, and activity in 10 dogs fed daily (BID), and intermittently fasted on a low fat (IF LF) and a high fat diet (LF HF) in a Latin Square design.

**Outcome**	**Diet**	**Mean**	**Standard deviation**	**Fixed effect**	**Estimate**	**Standard error**	***P*-value**
Intake	BID	295	40	(Intercept)	270	77	
(kcals/BWT^0.75^)	IF LF	230	49	Diet IF LF	−66	13	<0.0001
	IF HF	214	73	Diet IF HF	−81	12	<0.0001
				Sex male	51	17	0.027
Body weight	BID	−1.7	1.6	(Intercept)	−1.72	0.65	
(% change)	IF LF	−3.3	2.6	Diet IF LF	−1.56	0.85	0.067
	IF HF	−1.6	1.8				
Total activity (Δ G)	BID	254,058	78,988	(Intercept)	199,712	25,811	
	IF LF	227,637	77,512	Week	13,451	4,126	0.005
	IF HF	242,923	79,130				
Day activity (Δ G)	BID	212,251	69,033	(Intercept)	175,004	23,164	
	IF LF	200,763	72,634	Week	11,652	3,378	0.004
	IF HF	219,528	72,006				
Night activity (Δ G)	BID	39,458	15,615	(Intercept)	39,458	3,829	
	IF LF	28,261	8,872	Diet IF LF	−11,197	4,599	0.028
	IF HF	26,319	8,017	Diet IF HF	−13,665	4,771	0.012

*Also included are the results of the linear mixed effect models. Only independent variables with a P ≤ 0.1 are shown*.

### Metabolites

#### Metabolomics

**Figure 3:**
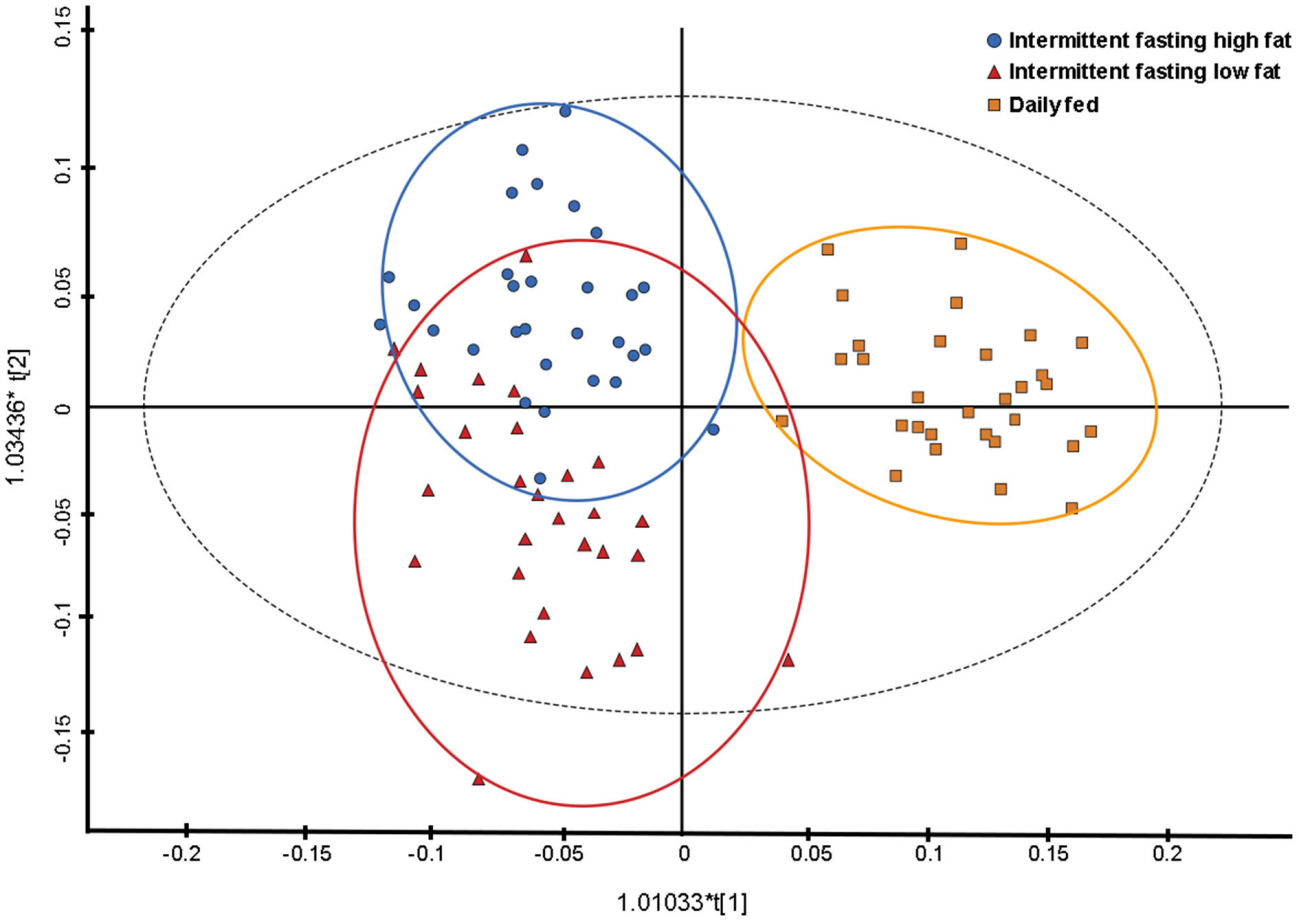
Orthogonal partial least square with discriminant analysis (OPLS-DA) plot illustrating the effect of three feeding regime on the plasma metabolome of 10 dogs. Each point represents a single sample with the blue circles representing a dog intermittently fasted on a high fat diet, the red triangles representing a dog intermittently fasted on a low fat diet, and the orange squares representing a dog fed daily on a low fat diet.

Principal component analysis indicated some separation between the diet groups, with the first two principal components accounting for 45.6 and 9.1% of the variance, respectively. Further analyses with a supervised orthogonal partial least squares discriminant model showed a complete separation of the daily fed group and the intermittently fasted groups, and clustering of the two intermittently fasted groups (Figure 3). The metabolites associated with the spectral buckets that separated the different feeding regimes the greatest were β-hydroxybutyrate, lactate, alanine and glucose.

#### β-Hydroxybutyrate Assayed via Kit and NMR

**Figure 4:**
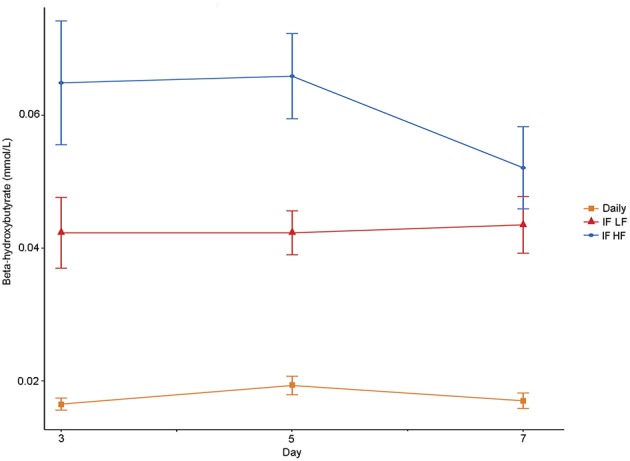
Mean concentrations (±SE) of fasted plasma beta-hydroxybutyrate in 10 dogs fed daily (square), and intermittently fasted on a low fat (triangle) and a high fat diet (circle) in a Latin Square design.

**Figure 5:**
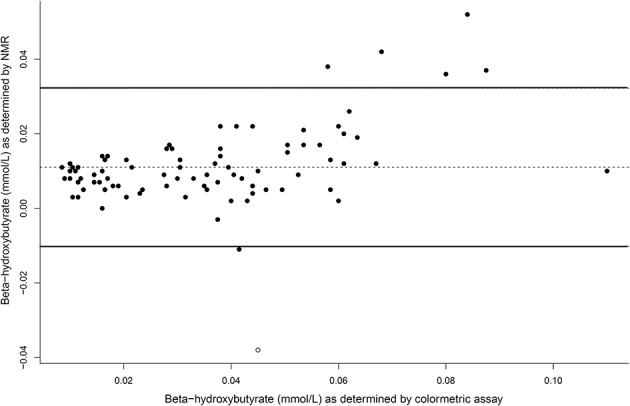
A Bland Altman comparison plot of beta-hydroxybutyrate concentrations as assay by the colormetric kit and by nuclear magnetic resonance (NMR). At y = 0, this indicates perfect agreement, with the middle line as the actual agreement. The solid lines represent the 95% limits of agreement of the data.

The concentrations of plasma β-hydroxybutyrate on day 3, 5, and 7 were highest when the dogs were intermittently fasted on the HF diet, and lowest when the dogs were fed daily (Figure 4). There was no effect of day. Intermittent fasting increased plasma β-hydroxybutyrate concentrations regardless of the diet fed, and a higher body condition score was associated with a decrease in plasma β-hydroxybutyrate (Table 3). There was no association between weight loss and plasma β-hydroxybutyrate concentrations (*P* = 0.198). There was reasonable agreement between the β-hydroxybutyrate concentrations obtained from the colorimetric kit and from NMR (Figure 5, Bland Altman).

**Table 3:** The means and standard deviations for β-hydroxybutyrate, lactate alanine and glucose, and the results of the linear mixed effect models, of 10 dogs fed daily (BID), and intermittently fasted on a low fat (IF LF) and a high fat diet (LF HF) in a Latin Square design.

**Outcome**	**Diet**	**Mean**	**Standard deviation**	**Fixed effect**	**Estimate**	**Standard error**	***P*-value**
β-hydroxybutyrate	BID	0.018	0.004	(Intercept)	0.115	0.026	
(mmol/L)	IF LF	0.043	0.013	Diet IF LF	0.028	0.003	<0.0001
	IF HF	0.061	0.024	Diet IF HF	0.043	0.003	<0.0001
				BCS	−0.020	0.004	<0.0001
Lactate	BID	0.652	0.150	(Intercept)	0.597	0.244	
(mmol/L)	IF LF	0.619	0.161	Day	−0.018	0.005	<0.001
	IF HF	0.571	0.163	Diet IF HF	−0.078	0.029	0.009
Alanine	BID	0.222	0.047	(Intercept)	0.246	0.057	
(mmol/L)	IF LF	0.221	0.036	Diet IF HF	−0.003	0.009	<0.001
	IF HF	0.191	0.042				
Glucose	BID	5.7	0.4	(Intercept)	5.9	0.5	
(mmol/L)	IF LF	5.6	0.3	Day	0.01	0.01	0.06
	IF HF	5.6	0.3	Diet IF LF	−0.12	0.06	0.03
				Diet IF HF	−0.13	0.06	0.02

*Only independent variables with a P ≤ 0.1 are shown*.

#### Lactate and Alanine via NMR

**Figure 6:**
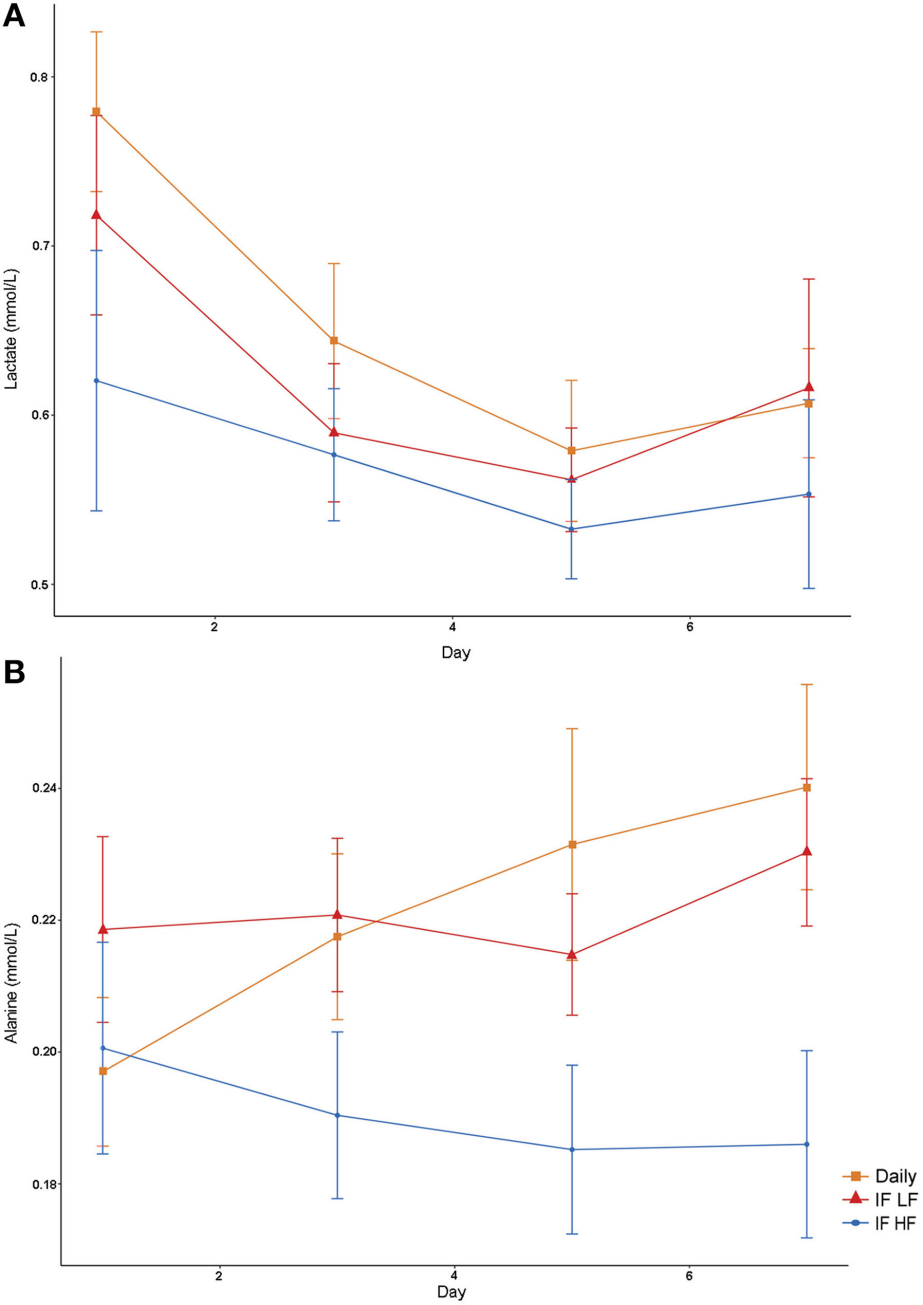
Mean concentrations (±SE) of fasted plasma lactate **(A)** and alanine **(B)** in 10 dogs fed daily (square), and intermittently fasted on a low fat (triangle) and a high fat diet (circle) in a Latin Square design.

Both lactate and alanine concentrations were lowest when the dogs were intermittently fasted on a high fat diet (Figure 6). In addition, lactate concentrations decreased over time (*P* = 0.009; Table 3).

#### Glucose

**Figure 7:**
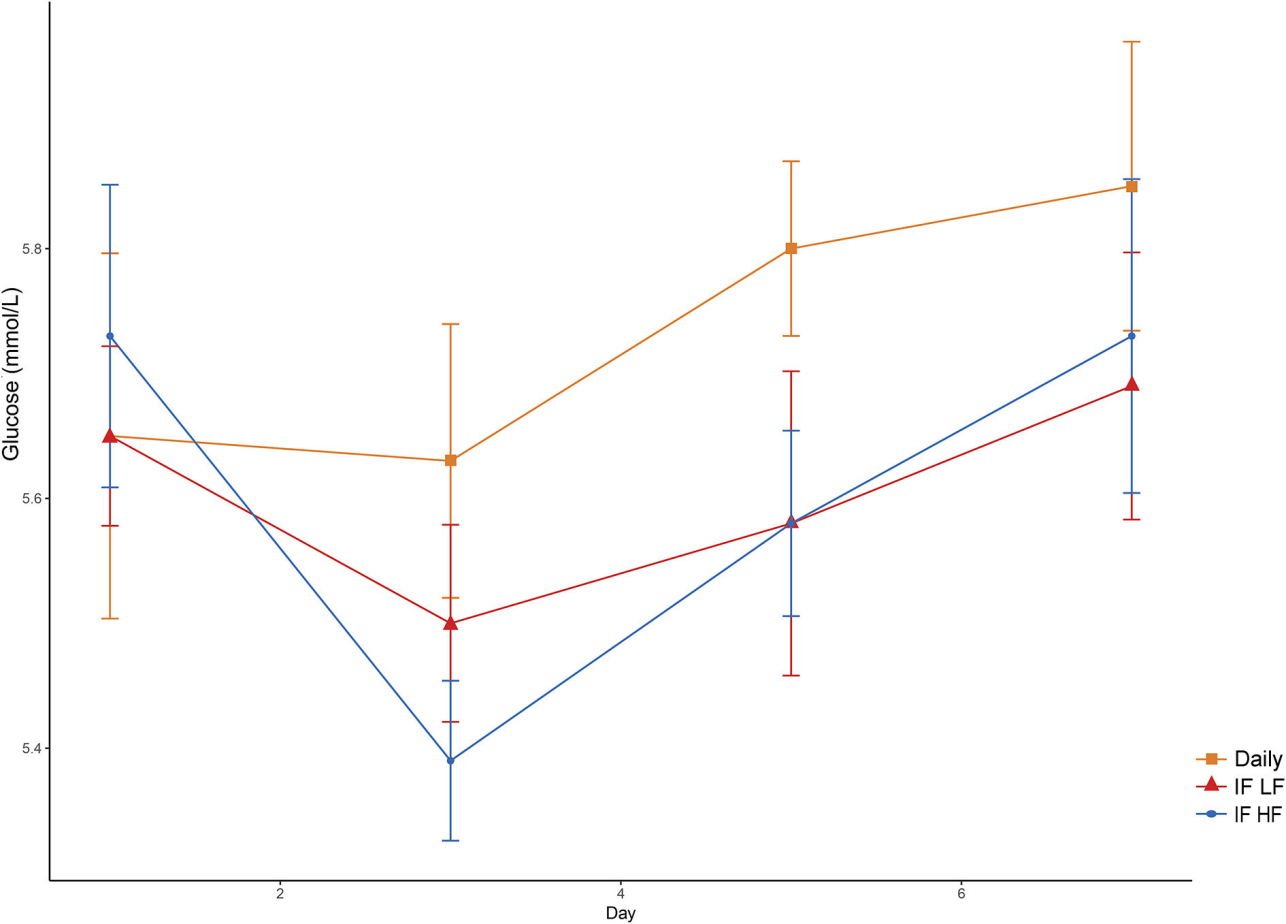
Mean concentrations (±SE) of fasted plasma glucose in 10 dogs fed daily (square), and intermittently fasted on a low fat (triangle) and a high fat diet (circle) in a Latin Square design.

Blood glucose concentrations increased over time from day 3 to day 7, and were highest in the dogs fed daily (Figure 7). There was no difference in glucose concentrations when the dogs were intermittently fasted on the LF and HF diets (*P* = 0.98). There was also no effect of BCS on blood glucose (*P* = 0.24; Table 3).

### Hormones Insulin, Leptin and Ghrelin, and HOMA

**Figure 8:**
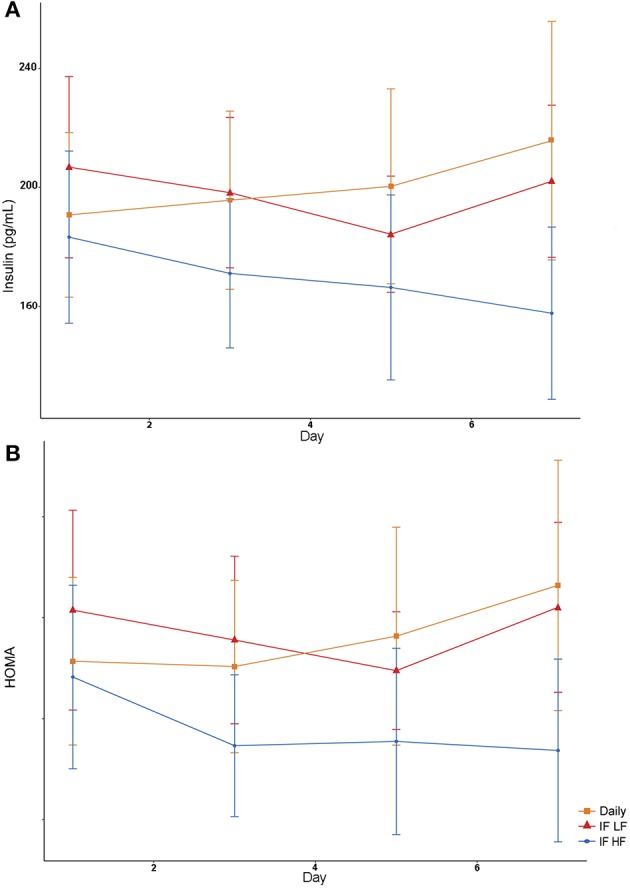
Mean concentrations (±SE) of fasted serum insulin **(A)** and HOMA score **(B)** in 10 dogs fed daily (square), and intermittently fasted on a low fat (triangle) and a high fat diet (circle) in a Latin Square design.

Both insulin concentrations and HOMA scores were lowest when dogs were intermittently fasted on a high fat diet (Figure 8). A higher body condition was associated with lower insulin concentrations and HOMA scores (Table 4).

**Table 4:** The means and standard deviations for insulin, Homeostatic Model Assessment (HOMA), leptin and ghrelin, and the results of the linear mixed effect models, of 10 dogs fed daily (BID), and intermittently fasted on a low fat (IF LF) and a high fat diet (LF HF) in a Latin Square design.

**Outcome**	**Diet**	**Mean**	**Standard deviation**	**Fixed effect**	**Estimate**	**Standard error**	***P*-value**
Insulin	BID	198	78	(Intercept)	449	123	
(pg/mL)	IF LF	200	101	Diet IF HF	−31.5	13.2	0.02
	IF HF	169	87	BCS	−44.1	19.6	0.03
Homeostatic	BID	1.46	0.63	(Intercept)	3.22	0.99	
Model Assessment	IF LF	1.45	0.76	Diet IF HF	−0.26	0.011	0.01
(HOMA)	IF HF	1.23	0.66	BCS	−0.32	0.15	0.04
Leptin	BID	2,451	2,217	(Intercept)	3,247	2,887	
(pg/mL)	IF LF	1,794	1,683	Day	−111	32	<0.001
	IF HF	1,729	1,433	Diet IF LF	−637	179	<0.001
				Diet IF HF	−743	179	<0.0001
Ghrelin	BID	85	78	(Intercept)	8.3	115	
(pg/mL)	IF LF	88	73	Day	6.6	1.4	<0.0001
	IF HF	67	60	Diet IF HF	−17.6	7.9	0.03

*Only independent variables with a P ≤ 0.1 are shown*.

**Figure 9:**
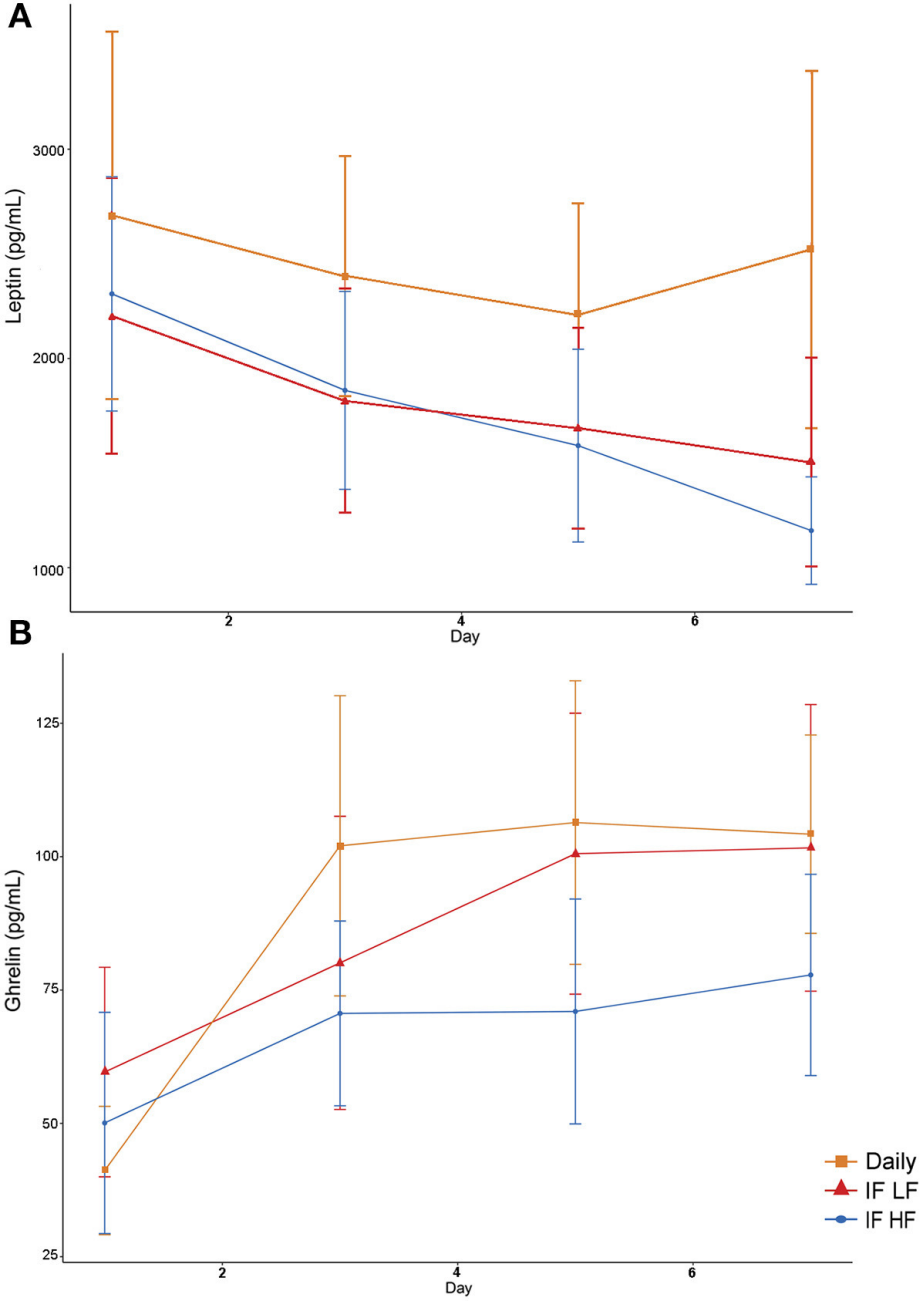
Mean concentrations (±SE) of fasted serum leptin **(A)** and ghrelin **(B)** in 10 dogs fed daily (square), and intermittently fasted on a low fat (triangle) and a high fat diet (circle) in a Latin Square design.

Serum leptin concentration was highest when dogs were fed daily (Figure 9). In addition, there was a decrease in leptin concentrations over time (Table 4). For ghrelin, dogs fasted intermittently on a HF diet had lower serum concentrations (Figure 9).

### Immunoassays

#### Lymphocyte Proliferation

Lymphocyte proliferation was not significantly different during any of the feeding regimes.

#### Phagocytosis and Respiratory Burst

**Figure 10:**
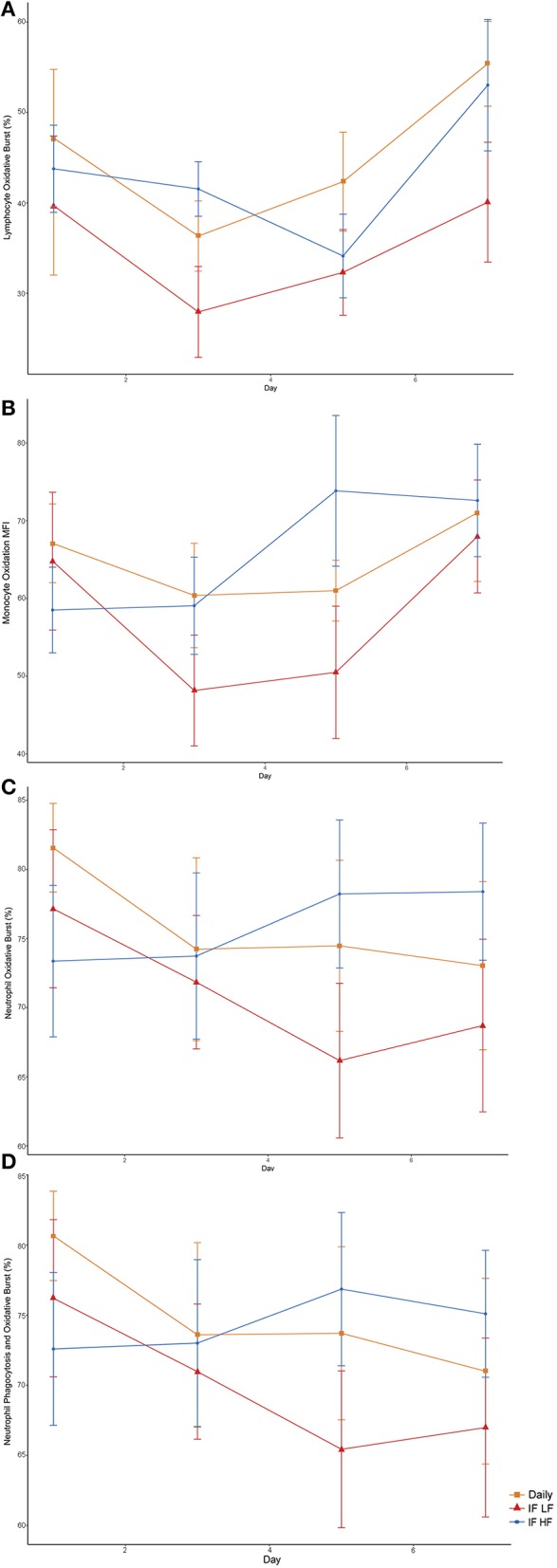
Differences in immune parameters in 10 dogs fed daily (square), and intermittently fasted on a low fat (triangle) and a high fat diet (circle) in a Latin Square design as assessed by flow cytometry. **(A)** Total percentage of lymphocytes which underwent oxidative burst. **(B)** The mean florescence intensity of oxidative burst in monocytes which underwent both phagocytosis and oxidative burst. This is a method of quantifying the degree of oxidation. **(C)** Total percentage of neutrophils which underwent oxidative burst. **(D)** The percentage of neutrophils which underwent both phagocytosis and oxidative burst.

When the dogs were intermittently fasted on a LF diet, they had a lower percentage of neutrophils, and a lower MFI in macrophages which underwent both phagocytosis and oxidation (Figure 10; Table 5). In addition, when on the IF LF feeding regime, the dogs also had a lower percentage of lymphocyte which underwent respiratory oxidative burst (Figure 10; Table 5).

**Table 5:** The results of the linear mixed effect models of the activity of phagocytic cells, and their respective means and standard deviations, in 10 dogs fed daily (BID), and intermittently fasted on a low fat (IF LF) and a high fat diet (LF HF) in a Latin Square design.

**Outcome**	**Diet**	**Mean**	**Standard deviation**	**Fixed effect**	**Estimate**	**Standard error**	***P*-value**
Lymphocyte oxidation	BID	45.3	18.4	(Intercept)	30.6	25.3	
(%)	IF LF	35.0	19.3	Diet IF LF	−10.7	3.9	0.008
	IF HF	43.1	17.1				
Monocyte phag+/ox+	BID	64.9	20.0	(Intercept)	72.3	28.6	
MFI for oxidation	IF LF	57.9	25.8	Diet IF LF	−7.6	4.0	0.06
	IF HF	66.0	23.5				
Neutrophil oxidation	BID	75.8	17.6	(Intercept)	73.6	23.3	
(%)	IF LF	71.0	17.6	Diet IF LF	−4.9	2.2	0.02
	IF HF	75.9	16.8				
Neutrophil phag+/ox+	BID	74.7	18.1	(Intercept)	67.9	23.2	
(%)	IF LF	69.9	17.6	Day	−0.8	0.4	0.04
	IF HF	74.4	16.4	Diet IF LF	−5.0	2.2	0.03

*Only independent variables with a P ≤ 0.1 are shown. The percentage is the number of cells that have undergone either phagocytosis and/or oxidative burst. Mean florescence intensity (MFI) of oxidation is proportional to the degree in which oxidation has occurred*.

## Discussion

Intermittent fasting and the promotion of ketone formation may be a beneficial feeding regime for hospitalized dogs, especially spinal patients. Our primary hypothesis was that healthy, intermittently fasted dogs would have lower fasting blood glucose, insulin and leptin concentrations, and greater fasting β-hydroxybutyrate and ghrelin concentrations compared to when they were eating daily. Our secondary hypothesis was that intermittently fasted dogs eating a high fat diet enriched with medium-chain triglycerides will have higher blood β-hydroxybutyrate and leptin concentrations compared with intermittently fasted dogs eating a LF diet. We found that dogs fasted for 48 h on a HF diet enriched in medium-chain triglycerides promoted higher blood β-hydroxybutyrate concentrations, and lower insulin concentrations than when they were fed daily or fasted on a LF diet. Leptin was not maintained during intermittent fasting by using a HF diet; however, there was no difference in the immune parameters studied between when the dogs were fed the HF diet and when they were daily fed. Fasted ghrelin concentrations were lowest when the dogs were intermittently fasted on a HF diet. Overall, these findings indicate that an intermittent fasting regime on a high fat diet enriched in medium-chain triglycerides increases plasma β-hydroxybutyrate concentrations without causing immune suppression, however it did not abrogate the drop in leptin or increase ghrelin concentrations during fasting.

A commercially available LF kibble was chosen in this study as the control diet. When dogs were eating the LF diet intermittently, they consumed fewer calories and lost more weight compared to when they were eating the HF diet. There are numerous studies in several species which also show this phenomenon (56). In one study, dogs fed *ad libitum* a high fat diet (51% energy from fat) gained more weight than dogs fed *ad libitum* on a low fat diet (23% energy from fat) (57). Therefore, to prevent weight loss in an intermittent fasting regime, it is likely that a high fat, energy dense food is required to ensure that a dog will consume its full requirements. However, there are epidemiological associations between high dietary fat consumption and pancreatitis in dogs (58). In early experimental models, dogs fed a high fat vs. a high protein/high carbohydrate diet led to more severe clinical signs and pathological changes in the pancreas (59, 60). However, the diets were unlikely to have been complete and balanced rations, and so other nutrient deficiencies may have confounded the results. Giving table scraps and consuming food discarded in the trash have also been identified as risk factors for the development of pancreatitis in dogs (61). However, it is difficult to speculate what the fat contents of those items were. In contrast, feeding diets with varying fat contents, including medium-chain triglycerides, to a group of healthy dogs did not have an effect on serum canine trypsin-like immunoreactivity (cTLI), pancreatic-lipase immunoactivity (cPLI), or gastrin concentrations (62). However, the highest fat diet in this study contained only ~40% fat on an energy basis. Still, there are numerous studies and reports of sled dog diets where the dogs were fed a diet with a similar fat content to our HF diet without causing pancreatitis (63–68).

Ketones provide an alternative source of energy for neurons and have been shown to reduce neuronal degeneration and improve recovery in rodent models of brain and spinal injury (30, 69–71). While β-hydroxybutyrate concentrations were highest in the dogs when they were intermittently fasted on the HF diet, the concentrations obtained (mean 0.061, SD 0.024 mmol/L) were much lower compared to rodents (0.8–2 mmol/L) and humans (1.67 mmol/L) fasted for a similar amount of time (22, 72, 73). Our finding is consistent with other published studies where the dog does not reach the same blood concentration of ketones as other species after comparable fasts (74–76). However, it has been shown that the rate of total ketone production is similar between dogs and men following a 48 h fast (77, 78). Further, De Bruijne and Van den Brom (74) established that dogs have a higher rate of clearance of plasma ketones than man. Thus, the seemingly low concentration of β-hydroxybutyrate in dogs is not from reduced production of ketones, but rather from higher rates of peripheral utilization compared with rodents and humans. In addition, although a single blood sample is indicative of the concentration of a metabolite at that moment, it does not describe its flux (production and utilization) (78). The concentration of β-hydroxybutyrate in the brain and cerebral spinal fluid (CSF) is proportional to the concentration found in plasma, and increases as the duration of fasting continues (72, 79, 80). When available, β-hydroxybutyrate is preferentially utilized over glucose, lactate and pyruvate by neurons as an energy substrate (81). So even a small increase in plasma concentrations of ketones could still provide a large contribution of energy for the brain and neurons.

In our study, all dogs had fasting blood glucose concentrations within the normal reference range, however lower values were obtained when the dogs were fasted for 48 h vs. 12 h. This reflects the difference in the length of fasting rather than the macronutrient composition of the diets. Similarly in a study of dogs eating either a carbohydrate-free or high carbohydrate diet, the blood glucose concentrations were the same after a 24 h fast in both groups of dogs (82). The lowest concentrations of insulin were seen when the dogs were intermittently fasted on the HF diet, which indicated a decrease in insulin production and/or increase in insulin sensitivity. The HOMA insulin resistance scores were also lowest when the dogs were on this feeding regime, which is a reflection of both low fasting glucose and insulin concentrations. Both caloric restriction and a reduction in fat mass improves insulin sensitivity in overweight dogs (83–86). In our study, intermittently fasted dogs eating a HF diet lost less weight than when intermittently fasted on a LF diet, and yet had lower insulin concentrations. Therefore, it appears that intermittent fasting on a high fat diet may reduce insulin production or improve insulin sensitivity independent of weight loss.

Alanine and lactate were two gluconeogenic metabolites identified by OPLS as being different between the feeding regimes. During the early stage of fasting, proteolysis of muscle releases alanine for gluconeogenesis in hepatocytes (87, 88). In a study of dogs eating a carbohydrate-free diet, the turnover rate of alanine and its conversion of alanine to plasma glucose were increased after a 48 h fast (89). Lactate is transported by the same monocarboxylate transporters (MCTs) as ketones, and also serve as an energy source for cells, including neurons, in a fasted state (90, 91). Feeding a high fat diet to rats increased the expression of MCT1 by brain endothelial cells (92). Also, lactate concentrations are increased in the brain of humans fasted for 2 days (72). Thus, the reduction of both alanine and lactate in the dogs when intermittently fasted on a HF diet may be due to an increase in uptake by the liver, brain, and kidneys.

The adipokine, leptin, has many roles in the body including the activation of phagocytosis by monocytes and chemotaxis of neutrophils and oxidative radical generation (93–95). In our study, we found that leptin concentrations were lowest in the intermittently fasted dogs regardless of the fat content of the diet. Both fasting and a reduction in fat mass are known to decrease leptin production (96, 97). During the course of our study, the dogs lost some weight; however, there was no difference in the starting concentrations of leptin at the beginning of each study week in any of the dogs. Therefore, the reduction in leptin concentrations was the result of the fasting regime, and not fat mass loss. In addition, although leptin was not maintained during fasting by feeding a HF diet, there was no difference between the immune parameters studied in those dogs and when the dogs were fed daily. We did find a reduction in the percentage of leucocytes undergoing phagocytosis and respiratory burst when the dogs were intermittently fasted on a LF diet. This suggests that the immune changes were not leptin-mediated. The suppressive effect however was not consistent throughout all the immune parameters studied, and the clinical significance of this reduction is not known.

### Limitations

Both diets used in this study were formulated to meet AAFCO requirements, and while all attempts were made to create similar nutrient profiles excluding the fat and carbohydrate content, the diets did differ from one another in some micronutrients. In addition, when the dogs were intermittently fasted on the LF diet, they lost more weight than in the other feeding regimes, indicating the dogs were in a greater catabolic state. However, an increase in proteolysis and fatty acid oxidation was not reflected by an increase in the plasma alanine and ketone concentrations of the dogs during the LF diet intermittent fasting regime. In addition, plasma ketone concentration was not associated with weight loss in this study. A washout week using the control feeding regime was performed in between each study period, and none of the outcome parameters were significantly different in the dogs at the start of each study period. Furthermore, diet order was examined in the multivariate model which did not show an effect. Our results suggest that the differences in diet profiles and greater weight loss during the LF diet intermittent fasting regime likely had a minimal effect, however, a more thorough study would be required to determine if that is indeed the case.

In our study, no ill effects were seen when the dogs were intermittently fasted on the high fat diet. Acute feeding of a high fat diet in other species can lead to an increase in circulating pro-inflammatory cytokines such as interleukin 1β and tumor necrosis-α, but this has not been shown consistently (98). Interestingly, an intermittent fasting regime has been shown to ameliorate the expression of proinflammation-related genes in hepatocytes during long-term high-fat feeding in mice (99). The effects of intermittent fasting on markers of inflammation during high fat feeding in dogs is not currently known. We did not measure inflammatory cytokines in our study, however future studies may wish to do so. Furthermore, a difference in immunity between the feeding regimes was apparent only when the dogs were intermittently fasted on the LF diet. The significance of this immune effect is not known, but there may be a greater implication of this in a clinical setting.

To promote ketone formation, coconut oil was used in the HF diet as a rich source of medium-chain triglycerides. However, the main medium-chain triglyceride constituent in coconut oil is dodecanoic acid (lauric acid, C12), with decanoic acid (capric acid, C10) and octanoic acid (caprylic acid, C8) as the great remainder (100). When given in equal amounts, intake of decanoic acid and octanoic acid leads to a greater ketone production postprandially then dodecanoic acid (101, 102). Thus, to increase the effect of medium-chain triglycerides on ketogenesis, a concentrated oil with a higher quantity of decanoic and octanoic acid can be given instead of coconut oil.

The homeostasis model assessment (HOMA) was developed to provide a measure of peripheral insulin resistance from fasting glucose and insulin. The scores correlate well with a euglycemic clamp model in humans, and has been used to detect improvements in insulin sensitivity with weight loss and fasting in humans (103–105). In our study, dogs intermittently fasted on a HF diet had the lowest HOMA score compared to when they were fed daily or intermittently fasted on the LF diet. However, HOMA has been found to be variably reliable in companion animals in detecting insulin resistance (106–108). So while we found a difference with HOMA scores in the different feeding regimes, any interpretation in peripheral insulin sensitivity should be confirmed using a euglycemic clamp.

## Conclusion

In this study, we found that fasting for up to 48 h in healthy dogs does not cause immunosuppression, and that fasting on a high fat diet enriched with medium-chain triglycerides promoted a greater plasma ketone concentration than when the dogs were fasted after eating a low fat diet. However, the concentrations obtained in dogs are significantly less than what is reported in other species fasted for a similar period of time. Therefore, a ketone kinetics study is required to gain a more comprehensive understanding of the flux of ketones in dogs while fasting for varying durations, and whether this feeding regime could be feasible in hospitalized dogs recovering from neuronal injury. In addition, dogs intermittently fasted on a low fat diet consumed fewer calories and lost more weight than when fed the high fat diet. Therefore, any practical application of this type of feeding regime would require modification of the diet itself (e.g., increase the energy density of the diet to reduce the volume and gut fill) and/or reducing the number of days or length of fasting. Ultimately, while we showed the possibility of this type of feeding regime to be feasible and to produce ketones without ill effect in healthy dogs, its application in hospitalized dogs remains to be determined.

## Data Availability Statement

The datasets generated for this study are available on request to the corresponding author.

## Ethics Statement

The animal study was reviewed and approved by Massey University Animal Ethics Committee (MUAEC #16/130).

## Author Contributions

YL and NC conceptualized and designed the study. YL was the primary researcher, with assistance from NC. YL organized data collection, completed laboratory preparation of blood samples, performed the statistical analysis, and wrote the first draft of the manuscript. AH supervised the immunological assays. PE performed the NMR analysis. AG reviewed the study design and statistical analysis. TW contributed by the reading and revision of the manuscript. All authors contributed to manuscript revision and approved the submitted version.

### Conflict of Interest

The authors declare that the research was conducted in the absence of any commercial or financial relationships that could be construed as a potential conflict of interest.

## Footnotes

^1^Eukanuba Large Breed Weight Control dry, Mars Incorporate, McLean, Virginia, USA.

^2^Balance IT® Canine, DVM Consulting Incorporated, Davis, California, USA.

^3^BD Vacutainer®, Plymouth, United Kingdom.

^4^Millipore® DPP-IV Inhibitor, Darmstadt, Germany.

^5^Heyrex®, Say Systems, Wellington, New Zealand.

^6^Accu-check® Performa, Roche Diagnostics Limited, Mannheim, Germany.

^7^beta HB Assay Kit, catalogue #ab83390, Abcam, Cambridge, United Kingdom.

^8^10kD spin column, catalogue #ab93349, Abcam, Cambridge, United Kingdom.

^9^VersaMax™ Microplate Reader, Molecular Devices LLC, San Jose, California, USA.

^10^Cryo.S™, Greiner Bio-One, Frickenhausen, Germany.

^11^DSS, Sigma-Aldrich, St. Louis, MI, USA.

^12^Imidazole, Sigma-Aldrich, St. Louis, MI, USA.

^13^5 mm NMR Sample Tubes, Wilmad-LabGlass, Vineland, New Jersey, USA.

^14^Milliplex® MAP Canine Gut Hormone Magnetic Bead Panel, Millipore, Burlington, Massachusetts, USA.

^15^Luminex 200™, Merck, Darmstadt, Germany.

^16^BD FASCSCalibur™, BD Sciences, San Jose, California, USA.

^17^pHodo™ Red *S. aureus* Bioparticles™, catalog number A10010, Invitrogen®, Carlsbad, California, USA.

^18^Dihydrorhodamine 123, catalog number D23806, Invitrogen®, Carlsbad, California, USA.

^19^BD Falcon™ tubes, BD Biosciences, San Jose, California, USA.

^20^FACSCalibur™, BD Biosciences, San Jose, California.
